# A Two-Hit Approach Inducing Flurothyl Seizures in Fmr1 Knockout Mice Impacts Anxiety and Repetitive Behaviors

**DOI:** 10.3390/brainsci14090892

**Published:** 2024-08-31

**Authors:** Katherine J. Blandin, David A. Narvaiz, Donald Gregory Sullens, Paige D. Womble, Samantha L. Hodges, Matthew S. Binder, Amanda Faust, Phuoc H. Nguyen, Zachary J. Pranske, Joaquin N. Lugo

**Affiliations:** 1 Department of Psychology and Neuroscience, Baylor University, Waco, TX 76798, USA; katie_blandin1@baylor.edu (K.J.B.); david_narvaiz1@baylor.edu (D.A.N.); paigedeannwomble@gmail.com (P.D.W.);; 2 Department of Pharmacology, University of Michigan Medical School, Ann Arbor, MI 48109, USA; hodgessamantha6@gmail.com; 3 Department of Neurosurgery, Yale University School of Medicine, East Haven, CT 06520, USA; mbinder@trinity.edu; 4 Department of Neuroscience, University of Maryland, Baltimore, MD 20742, USA; phuoc.nguyen@som.umaryland.edu; 5 Department of Biology, Brandeis University, Waltham, MA 02453, USA; zpranske@brandeis.edu; 6 Department of Biology, Baylor University, Waco, TX 76798, USA; 7 Institute of Biomedical Studies, Baylor University, Waco, TX 76798, USA

**Keywords:** epilepsy, mTOR, early life seizures, two-hit, double-hit, developmental epilepsy

## Abstract

Background: Fragile X Syndrome (FXS) is the leading monogenetic cause of autism spectrum disorder (ASD) and is associated with seizures. We examined the impact of repeated seizures on the behavioral and molecular changes in male Fmr1 knockout (KO) mice and wild-type (WT) mice. Methods: Seizures were induced by administering three flurothyl seizures per day across postnatal days (PD) 7–11, for a total of 15 seizures. In adulthood, mice were tested in a battery of behavioral tasks to assess long-term behavioral deficits. Results: The two-hit impact of a Fmr1 knockout and seizures resulted in decreased anxiety-like behavior in the elevated plus maze test and a longer latency to their first nose poke (repetitive behavior). Seizures resulted in decreased activity, decreased repetitive behavior (grooming and rearings), and decreased social behavior, while they also increased habituation to auditory stimuli and increased freezing in delayed fear conditioning in both KO and control mice. KO mice displayed increased repetitive behavior in the open field task (clockwise revolutions) and repeated nose pokes, and decreased anxiety in the open field test. No differences in mTOR signaling were found. Conclusions: These findings further illuminate the long-term effects of synergistic impact of two hits on the developing brain.

## 1. Introduction

Fragile X Syndrome (FXS) is a neurodevelopmental disorder affecting 1 in 7000 males and 1 in 11,000 females worldwide [1], serving as the leading cause of inherited intellectual disability [2]. The disorder is caused by the silencing of the Fragile X Messenger Ribonucleoprotein 1 gene (FMR1) through the expansion of a CGG trinucleotide sequence on the X chromosome. In humans, FXS results in symptoms impacting locomotor activity, language, executive functioning, hyperactivity, stereotypic behavior, and anxiety [3]. Mutations in Fmr1 are the leading monogenic cause of autism spectrum disorder (ASD), accounting for 2–5% of those with ASD [4], resulting in an overlap of symptoms in both behavioral phenotypes. Seizures are the most common secondary medical diagnosis in those with FXS [5], with epilepsy affecting 10–20% of those with FXS [6].

The average age of FXS diagnosis is around three years, leading researchers to speculate whether early life insults contribute to long-term behavioral and cognitive outcomes [7]. It is well-established that early-life seizures can lead to cognitive deficits in working memory and executive control [8,9,10,11]. A question that remains is whether superimposing seizures on FXS potentiates the development of these cognitive deficits. Superimposing seizures on a genetic model of autism can be a helpful model to evaluate how seizures and a genetic predisposition to ASD interact and lead to behavioral deficits. Previous findings have shown a link between seizure frequency and changes in ASD-like symptoms [12]. 

Elevated mTOR activity has been consistently associated with neurological disorders relating to ASD, epilepsy, and neurodegenerative disorders [13]. The mammalian target of rapamycin (mTOR) pathway may play a vital role in the disruption of FMRP levels [13]. An increase in mTOR signaling can also result in alterations in dendritic ion channel levels, microglia activation, and astrogliosis levels [14,15,16]. Elevated phosphorylation of S6, a kinase that is downstream of the mTOR cascade, in the cortex of Fmr1 knockout (KO) mice has been correlated with increased audiogenic seizure susceptibility [17]. Upregulation of FMRP-mediated protein synthesis has been partially rescued in Fmr1 KO mice when S6 has been genetically deleted. Rescuing hyperactive mTOR expression has been shown to mitigate behavioral deficits associated with FXS [18]. 

ASD is a heterogeneous disorder, and its etiology can be complicated to disentangle from the impact of other neurological insults experienced in early development. By using a two-hit experimental design, one can investigate the impact of each insult and the result of superimposition of both insults (genetic deletion of Fmr1 and early life seizures). For this study, we investigated the behavioral and molecular effects of early-life seizures induced from postnatal day (PD) 7–11 of male Fmr1 wild-type (WT) and KO mice to determine the impact of superimposing an insult on a monogenetic condition. We then examined the behavioral effects of early-life seizures on the genetic condition through a battery of behavioral tests that measured changes in locomotor activity, stereotypic behavior, anxiety-like behavior, repetitive behavior, sensorimotor gating, and learning. We then examined changes in the mTOR pathway by using Western blotting of the hippocampal tissue.

## 2. Materials and Methods

### 2.1. Animals

Female heterozygous (+/−) mice were bred with male wildtype (+/+) mice to produce male wildtype (+/+) mice (WT) and male knockout (−/−) mice (KO). Since this is an X-linked gene, male mice lacking the gene on their X chromosome are considered a full knockout. All mice were housed and bred at Baylor University in a temperature-controlled colony room maintained at 22 °C. The mice were kept on a 12 h light and 12 h dark diurnal cycle, and all animals had access to food and water ad libitum. Testing and seizure induction took place during the light phase between 8 am and 5 pm each day. The beginning sample size for animals is as follows: N Total = 54 (Control, wildtype = 24; Control, knockout = 10; Seizure, wildtype = 10; Seizure, knockout = 10). The sample size was determined by a power analysis. For Western blotting, tissue was collected from 8 mice per group. Behavior testing began at 3 months of age, starting with the least invasive task and ending with the most invasive task in the following order of events: open field, elevated plus maze, nose poke, social partition, delay fear conditioning, and pre-pulse inhibition. This was done to minimize test-to-test effects [19]. Each task was run no less than 24 to 48 h after the previous task. All procedures were approved by the Baylor University Institutional Care and Use Committee and followed the Guide for the Care and Use of Laboratory Animals of the National Institutes of Health.

### 2.2. Seizure Induction

On postnatal day (PD) 7, mice were randomly assigned to either the control or seizure group and then clipped for identification based on genotype. All mice were then returned to their home cages for 30 min before any seizures were induced. Beginning on PD 7 through PD 11, the chemoconvulsant flurothyl (bis-2,2,2-trifluoromethyl ether) supplied by Sigma Aldrich (St. Louis, MO, USA) was utilized to induce a series of three generalized tonic–clonic seizures, with a 2 h recovery period between each occurrence. Control- and seizure-group litters were made up of two to three mice from the same home cage. The litters were placed in a clear acrylic inhalation chamber (29 cm × 16 cm × 15 cm) within a fume hood where flurothyl was administered via inhalation utilizing a Harvard Apparatus syringe pump (Model 11 Plus) dispensed at a rate of 50 µL/minute. Mice were kept in the chamber until all the mice reached tonic–clonic seizure status (tonic extension of forelimbs and hindlimbs) [9]. Control mice were placed in an identical inhalation chamber for the same duration their yoked seizure counterparts required to achieve tonic–clonic seizures. 

### 2.3. Open Field

The open field was used to examine locomotor activity, repetitive behavior, and anxiety-like behavior as previously described [20]. Mice were weighed and their tails were marked before a 30 min acclimation period. For the test, mice were placed in the center of a clear acrylic arena (41 cm × 41 cm × 32 cm) with a white base in the testing room with lighting (100 lux). The experimenter left the testing room for the 30 min test duration, and all behavioral measures were recorded and scored using Fusion (version 4.5; Omnitech, Columbus, OH, USA). 

### 2.4. Elevated Plus Maze

An elevated plus maze was used to assess anxiety-like behavior [20]. The elevated plus maze (40 cm above the floor) is made of four arms (30 × 5 cm each), with two opposing arms open and the other two arms having acrylic sides (15 cm). After a 30 min acclimation period, the subjects were placed in the center of the apparatus so that the mice had a view of the open arms. Mice were left to explore the apparatus for 10 min without the experimenter present. 

### 2.5. Nose Poke 

A nose poke assay is used to assess repetitive behavior [21]. Mice were placed in the center of a clear acrylic arena (41 cm × 41 cm × 28 cm) containing 16 equidistant holes (diameter 1.5 cm) spaced 9 cm apart on the floor of the arena. Mice were given 10 min to explore the arena, and scoring was divided into 5 min intervals. 

### 2.6. Social Partition

Social partition was used to observe social behavior based on frequency and duration spent at the partition [20]. Mice were housed in a standard cage evenly separated using an acrylic divider with 0.6 cm diameter holes and matched with the age, sex, and weight of the conspecific C57BL/6J mouse on the other side of the partition. After 24 h, the mice were tested in three sequential 5 min trials as follows: trials 1 & 3, familiar mouse (partner paired with experimental subject overnight for 24 h); trial 2, unfamiliar (novel) mouse. 

### 2.7. Delay Fear Conditioning

Delay fear conditioning (FC) was used to assess contextual and cued memory [20]. There were three phases to the FC protocol: training, context test, and cued response test. All procedures took place in a fear chamber (Coulbourn Habitest^®^, Coulbourn Instruments, Holliston, MA, USA) with shock-grid flooring, two clear plexiglass walls, and two metal walls inside a sound-dampening chamber (Coulbourn Habitest^®^, Coulbourn Instruments). 

### 2.8. Pre-Pulse Inhibition

Pre-pulse inhibition was used to measure acoustic startle response for sensorimotor gating through habituation, pre-pulse inhibition (PPI), and startle response. The mice were placed in a hollow acrylic restraint (3 1⁄2” (L) × 1.1” (inner diameter)), which is secured on the sensor platform within the testing chamber. The SR-LAB Startle Response System (San Diego Instrument, San Diego, CA, USA) elicited and detected startle responses at a certain amplitude. Over a week, a three-day PPI protocol was used to detect the sensorimotor gating response as previously described [21]. 

### 2.9. Western Blot Analysis

Western blotting was used to assess changes in inflammatory markers as well as mTOR pathway protein levels due to seizures. Mice were euthanized by rapid decapitation at five months of age. The hippocampus was dissected, rinsed with 1× PBS, set on dry ice, and then stored at −80 °C until it was ready to be processed. For processing, the entire hippocampal tissue was placed in a homogenization buffer solution (0.32 M sucrose, 1 mM EDTA, and 5 mM Hepes) that also included a protease and phosphatase inhibitor cocktail (Sigma, St Louis, MO, USA), producing a homogenate. The homogenate was then centrifuged at 4 °C for 1 min at 1000× *g*. The supernatant was then removed and stored in a separate tube and the pellet was discarded. One hundred microliters of the supernatant were then removed for the total hippocampal sample, and the remaining supernatant was centrifuged for 10 min at 800× *g* for the P2 sample of supernatant for synaptosome processing. The pellet was used as the P1 sample and 200 µL of PIC homogenizing buffer was used to resuspend the sample. To complete the P2 sample, the supernatant was centrifuged at 7200× *g* for 15 min and the supernatant was examined for synaptosome fraction. Proteins to investigate epilepsy-related neuroinflammation were glial fibrillary acidic protein (GFAP) for tagging astroglia and ionized calcium-binding adaptor molecule 1 (Iba1) for microglia activation. Potassium/sodium hyperpolarization-activated cyclic nucleotide-gated channels 1 & 2 (HCN1 & HCN2) and potassium voltage-gated Shal subfamily 2 (Kv4.2) were measured for the analysis of neuron excitability, commonly dysregulated in the presence of repeated seizures. Synaptic transmission regulation through PSD-95 was examined as commonly associated with decreased FMRP levels. To assess the mTOR signaling pathway, ribosomal protein S6 (S6) and protein kinase B (AKT) were measured. All proteins were percent-controlled to actin.

### 2.10. Statistical Analysis

All statistical analyses were completed using SPSS (version 23), and all graphs were created using GraphPad (Prism 7). The analyses for open field, elevated plus maze, nose poke, delay fear conditioning day 2a, and Western blot were done using two-way ANOVA (Genotype [WT, KO] × Treatment [Control (CTL), Seizure]). From the two-way ANOVA, any significant interactions found were then analyzed further using separate *t*-tests. We could not use post hoc tests since there were only two groups for treatment and two groups for genotype. Post hoc analyses are not allowed when there are fewer than two groups. For social partition, a two-way repeated-measures ANOVA was done to measure the frequency and duration at the partition with a within-subjects factor by trial (familiar, unfamiliar, familiar), with significant interactions between subjects being assessed with independent *t*-tests. 

For significant within-subjects effects, a trial-specific univariate ANOVA was run to find significance within the groups in significant trials. Pre-pulse inhibition habituation and the startle threshold were also analyzed using a two-way repeated-measures ANOVA within the subjects’ factor of trial block for habituation and intensity for the startle threshold. Day 1 for delay fear conditioning habituation was also analyzed by two-way repeated-measures ANOVA with a within-subjects factor comparison of the trial (baseline, tone trial 1, inter-trial interval 1, tone trial 2, inter-trial interval 2). Animals that were included in the statistical analysis were tested in at least one behavioral task. The level of statistical significance was set to be at *p* < 0.05, and data are presented in the form of mean ± the standard error of the mean. Since there were only two groups for treatment and two groups for genotype, we ran separate *t*-tests to evaluate differences between groups.

For the results below, we only included the results that were statistically significantly different due to space constraints and to improve clarity. We have included the full statistics as a supplemental file (Supplemental S1) to this manuscript.

## 3. Results

### 3.1. Early-Life Seizures Decreased Locomotor and Stereotypic Behavior but Did Not Impact Anxiety-like Behavior in the Open Field

**Figure 1:**
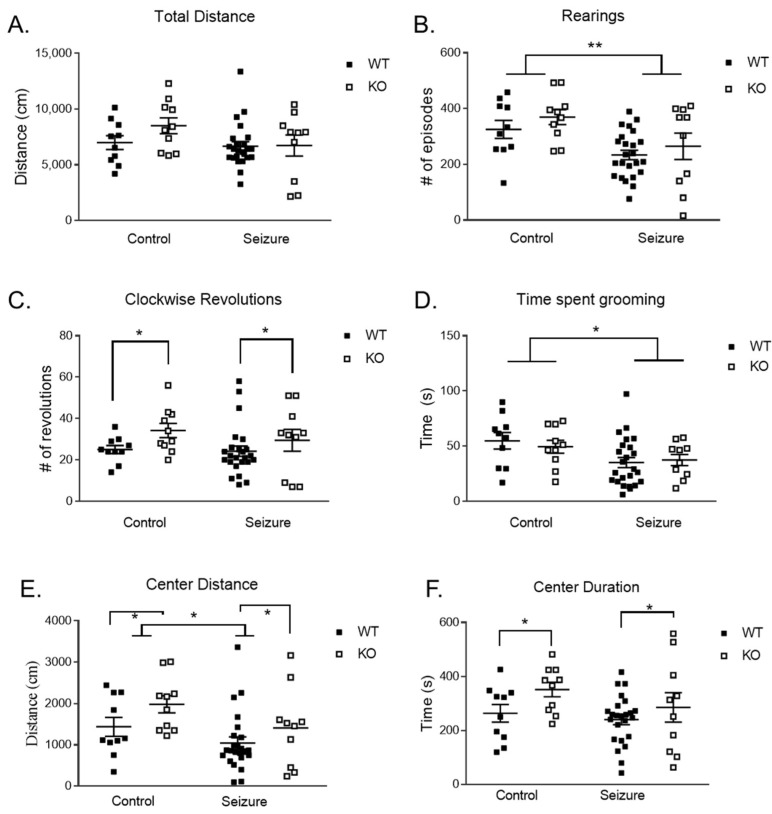
**Open field test.** There was no effect of treatment or genotype on total distance traveled in the open field (**A**). The number of rearings was reduced in Fmr1 wildtype (WT) and knockout (KO) mice that had seizures (**B**). Fmr1 KO mice displayed increased clockwise revolutions compared to WT mice, with no effect of treatment (**C**). The time spent grooming was reduced in WT and KO mice that received seizures. (**D**). There was an increase in distance traveled in the center portion of the open field for Fmr1 KO mice compared to WT mice, as well as a reduction in distance traveled in the center in the Fmr1 KO and WT mice that received seizures. (**E**). The time spent in the center portion of the open field test was greater in Fmr1 KO compared to WT mice (**F**). Data are expressed as mean ± standard error of the mean (SEM), ** = *p* < 0.01, * = *p* < 0.05. Each square represents a single subject. The WT mice are in solid squares and the KO mice are in open squares.

The open-field test was used to assess exploratory behavior and locomotor activity. There was no difference in total distance (Figure 1A). Early-life seizures resulted in significantly less rearing in the open field test [F (1, 50) = 10.90, *p* < 0.01] (Figure 1B). For the number of clockwise revolutions to measure repetitive behavior, there was a main effect of genotype [F (1, 50) = 4.13, *p* < 0.05] with the KO mice performing significantly more revolutions compared to WT mice (Figure 1C). We observed a main effect of treatment for time spent grooming to measure stereotypic behavior [F (1, 50) = 6.78, *p* < 0.05], such that animals that experienced seizures had a reduction in stereotypy time compared to controls. 

Anxiety-like behaviors were also measured using the open-field task, using the total distance that the mouse moved in the center of the arena of the testing apparatus. For the distance traveled in the center of the arena, there was a main effect of treatment [F (1, 50) = 4.76, *p* < 0.05], with the mice in the seizure group traveling shorter distances in the center compared to controls (Figure 1E). Genotype also had a significant effect on distance traveled in the center [F (1, 50) = 4.23, *p* < 0.05], with KO mice having moved more in the center than WT. There was a main effect of genotype on the duration in the center [F (1, 50) = 4.16, *p* < 0.05], with KO mice spending more time in the center compared to WT (Figure 1F). 

### 3.2. Early-Life Seizures Decreased Locomotor Activity but Did Not Affect Anxiety-like Behavior in the Elevated Plus Maze

**Figure 2:**
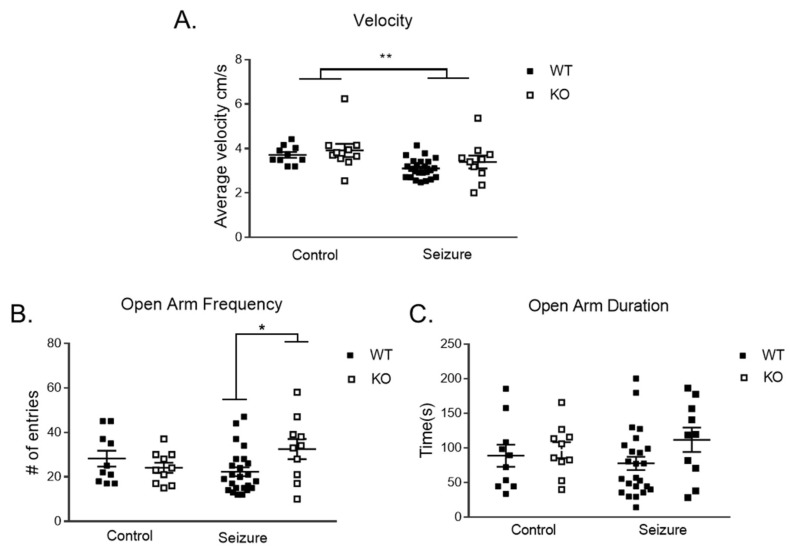
**Elevated plus maze**. Fmr1 knockout (KO) and wildtype (WT) mice that received seizures had a reduction in velocity in the elevated plus maze compared to controls (**A**). Early-life seizures increased in the number of times the Fmr1 KO mice entered the open arm in the elevated plus maze test compared to WT mice that received seizures (**B**). There was no difference in the time spent in the open arm for Fmr1 WT or KO control and seizure mice (**C**). Data are expressed as mean ± standard error of the mean (SEM), * = *p* < 0.05, ** = *p* < 0.01. Each square represents a single subject. The WT mice are in solid squares and the KO mice are in open squares.

Locomotor activity through velocity was analyzed utilizing a two-way ANOVA, showing the main effect of treatment [F (1, 50) = 8.81, *p* < 0.01], with seizure mice traveling slower compared to controls (Figure 2A). As a test for anxiety-like behavior, we analyzed the frequency of entrances and duration in the open arms to suggest a less anxious phenotype using the elevated plus maze. For the frequency in the open arms, there was a two-way interaction of treatment and genotype [F (1, 50) = 5.29, *p* < 0.05] (Figure 2B). With this significant interaction, we followed up with a *t*-test and found a significant increase in frequency in the open arm in the Fmr1 KO/seizure compared to Fmr1 WT/seizure t (1, 32) = 2.41, *p* < 0.05. There were no effects on the time spent in the open arm (Figure 2C). 

### 3.3. Early-Life Seizures Decrease Exploratory Behavior in Fmr1 KO Mice in the Nose Poke Task

**Figure 3:**
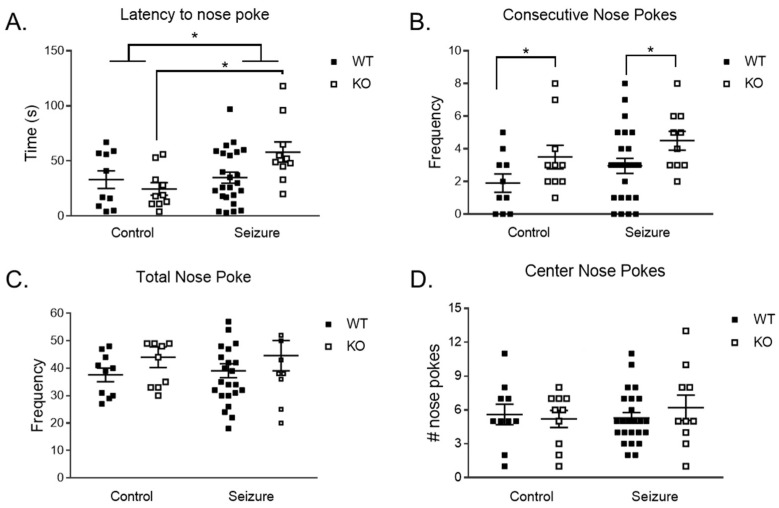
**Nose poke assay**. There was an interaction between treatment and genotype for the latency to the first nose poke. The Fmr1 KO mice with early-life seizures had a longer latency to engage in their first nose poke compared to the Fmr1 KO control mice. There was also an increase in latency to nose poke for the WT and KO mice that had seizures compared to the control WT and KO mice (**A**). Fmr1 KO mice exhibited an increased number of total nose pokes compared to WT mice, with no effect of treatment (**B**). There were no differences in the total number of nose pokes (**C**) or center nose pokes (**D**) between any of the conditions. Data are expressed as mean ± standard error of the mean (SEM), * *p* = < 0.05. Each square represents a single subject. The WT mice are in solid squares and the KO mice are in open squares.

We used a hole board to assess nose poking behavior as a measure of repetitive behavior. A two-way ANOVA detected a main effect of treatment on latency to the first nose poke [F (1, 50) = 6.02, *p* < 0.05], with the seizure group showing a longer latency to the first nose poke (Figure 3A). There was a significant interaction between genotype and treatment for the latency to the first nose poke [F (1, 50) = 4.85, *p* < 0.05]. With this significant interaction, the animals were subdivided into four groups for a post hoc analysis: Fmr1 WT/Control, Fmr1 KO/Control, Fmr1 WT/seizure, and Fmr1 KO/seizure. The multiple comparisons showed that seizures in KO mice had a longer delay in time to the first nose poke by comparison to control KO mice (*p* < 0.05).

When looking at the number of times a mouse did consecutive nose pokes as a means of repetitive behavior, a two-way ANOVA revealed a main effect of genotype on the number of consecutive nose pokes [F (1, 50) = 6.48, *p* < 0.05], with KO mice nose poking more often compared to WT (Figure 3B). As another measure of repetitive behavior, we also looked at the total number of nose pokes in each group (Figure 3C) and the number of center nose pokes (Figure 3D). We found no main effects of interactions in the last two measurements. 

### 3.4. Early-Life Seizures Reduce Social Behavior in the Social Partition Task

**Figure 4:**
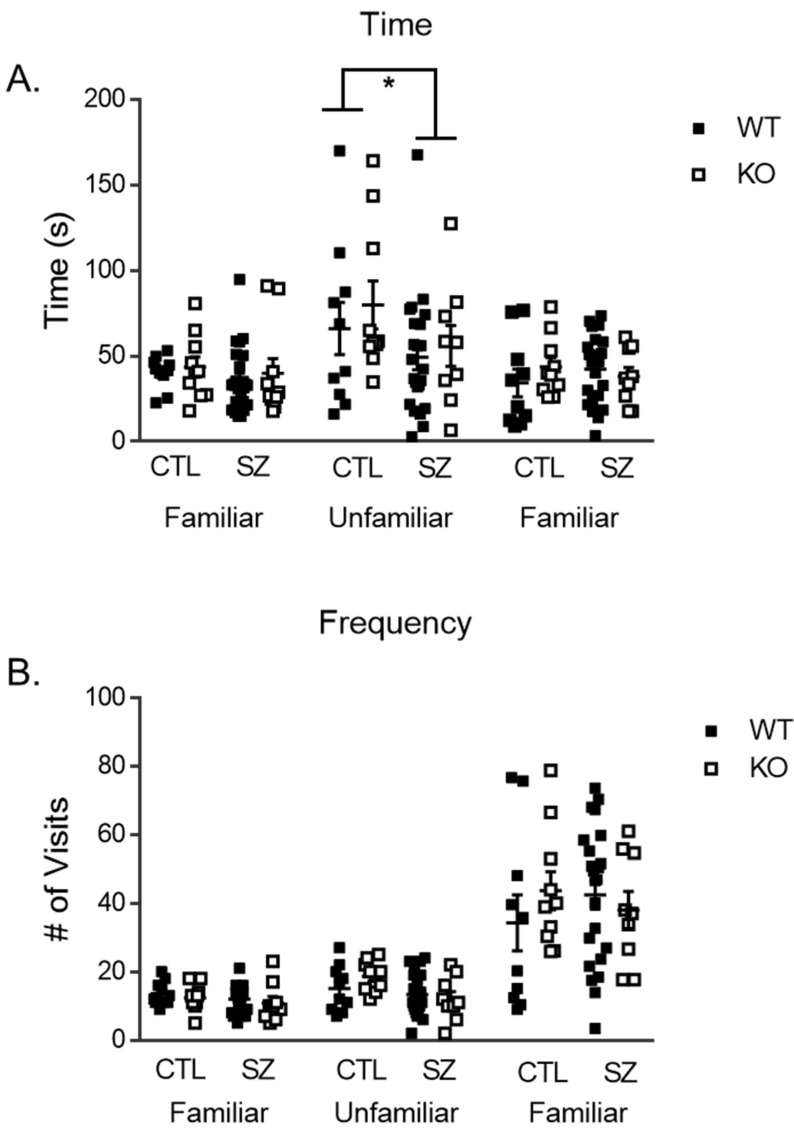
**Social partition task.** Seizure mice spent less time at the partition compared to control mice in the unfamiliar trial with no effect of genotype (**A**). There was no difference in the number of visits to the partition in any of the trials. (**B**). Data are expressed as mean ± standard error of the mean (SEM), * *p*= < 0.05. Each square represents a single subject. The WT mice are in solid squares and the KO mice are in open squares.

To assess changes in social behavior, a two-way repeated-measures ANOVA was used to analyze the social partition task. Changes across trials (1: familiar mouse, 2: unfamiliar, 3: familiar) were examined. One WT/seizure mouse was excluded as the data were not properly collected. The within-subjects comparison with a factor of “trial” showed that there was an effect of treatment and duration spent at the partition over the trials [F (1,49) = 3.53, *p* < 0.05], but no effect of genotype and duration [F (1, 49) = 0.34, *p* = 0.71] (Figure 4A). Following further analysis with independent *t*-tests for treatment in each trial, however, there was no significant effect within each of the three trials. There was a significant difference between treatment on the unfamiliar task [t (1, 51) = 2.1, *p* < 0.05]. The seizure mice showed a reduction in time spent with the unfamiliar mouse. There was no two-way interaction between treatment, genotype, and duration spent at the partition across trials [F (1, 49) = 0.48, *p* = 0.62]. A between-subjects analysis showed no significant main effects of genotype [F (1, 49) = 0.34, *p* = 0.57], treatment [F (1, 49) = 2.01, *p* = 0.16], or interaction of treatment and genotype on the amount of time spent at the partition [F (1, 49) = 0.57, *p* = 0.46]. We found no differences in the frequency of visits to the social partition (Figure 4B). We found no differences in treatment, genotype, or interaction.

### 3.5. Early-Life Seizures Increase Habituation and Startle Response in the Pre-Pulse Inhibition Task

**Figure 5:**
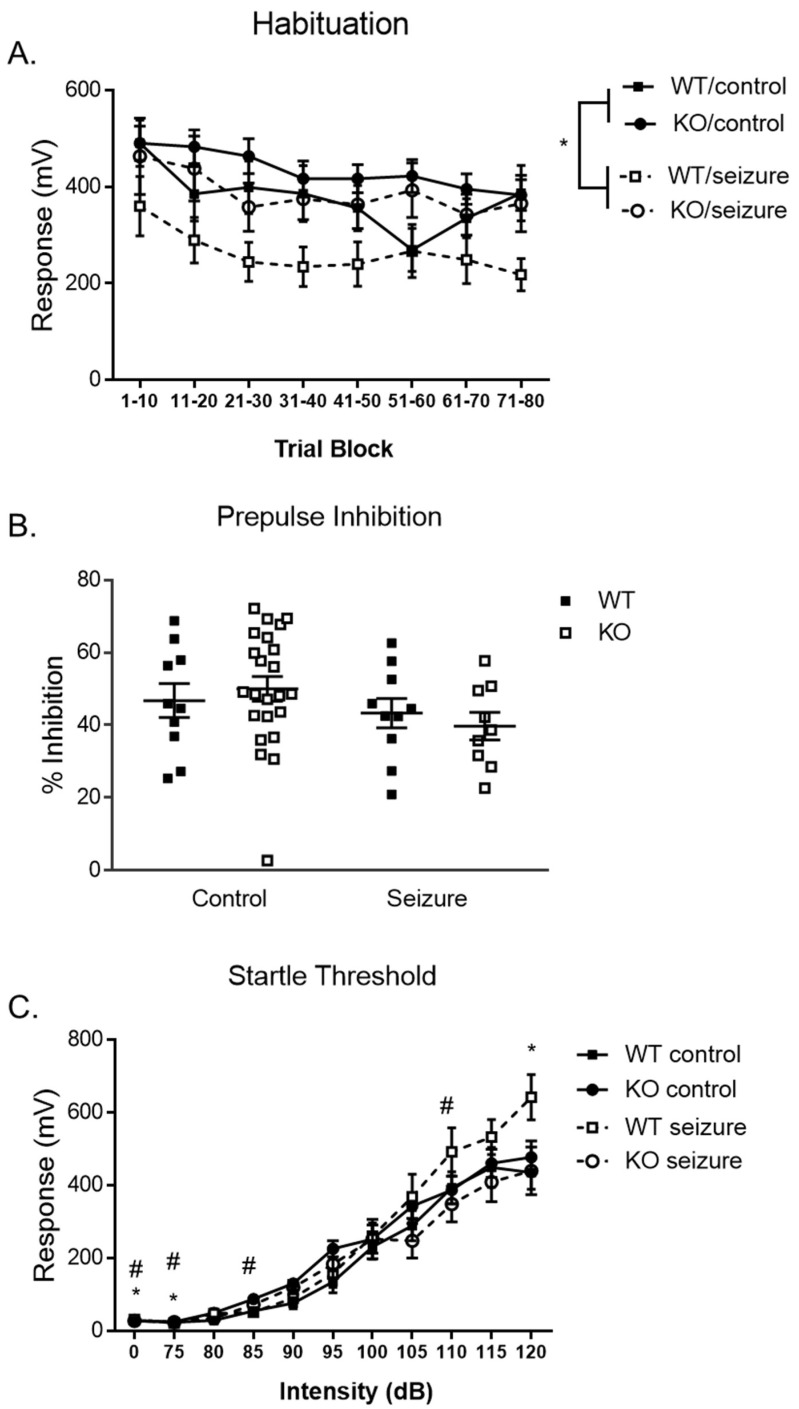
**Habituation, pre-pulse inhibition, and startle threshold**. During the habituation trials, the Fmr1 wildtype (WT) and knockout (KO) mice demonstrated greater habituation across the 80 trials compared to control mice (**A**). There was no difference in the percent inhibition in the pre-pulse inhibition assay (**B**). There were differences in WT and KO mice and differences in control and seizure mice for the startle response (**C**). * denotes a seizure effect at *p* < 0.05 on the graph and # represents a significance level of *p* < 0.05 for a genotype effect. Each square represents a single subject. The WT mice are in solid squares and the KO mice are in open squares.

Sensorimotor gating through the startle response was examined using our pre-pulse inhibition (PPI) test, starting on day 1 with the habituation of the stimulus stage. Mice were habituated to the testing apparatus for 5 min with startle stimuli presented every 15 s. A two-way repeated-measures ANOVA and a within-subjects factor of “time” (80 startle stimuli were grouped into 10 trials to create 8 time period bins). There was a significant interaction of treatment over trials [F (7, 49) = 2.5, *p* < 0.05] (Figure 5A). When we ran follow-up *t*-tests, we found significant differences at the 11–20 [t (1, 51) = 2.7, *p* < 0.01], 21–30 [t (1, 51) = 2.7, *p* < 0.01], 31–40 [t (1, 51) = 2.7, *p* < 0.01], 41–50 [t (1, 51) = 3.0, *p* < 0.01], 51–60 [t (1, 51) = 3.4, *p* < 0.001], 61–70 [t (1, 51) = 3.0, *p* < 0.01], and 71–80 bins [t (1, 51) = 2.1, *p* < 0.05]. The between-subjects effects for the time bins revealed a main effect of treatment [F (1, 49) = 6.79, *p* < 0.05], with those mice that received seizures expressing a greater level of habituation overall in that the response to the startling auditory stimuli became reduced over time. 

The second day of PPI testing began 24 h after the habituation day, measuring pre-pulse inhibition. We used a two-way ANOVA for percent inhibition and found no significant main effects of treatment [F (1, 49) = 0.01, *p* = 0.94] or genotype [F (1, 49) = 2.45, *p* = 0.12] on the percent pre-pulse inhibition (Figure 5B). No interaction of treatment and genotype was found [F (1, 49) = 0.57, *p* = 0.45]. 

One week following, the startle threshold was measured on the third day of testing. A two-way repeated-measures ANOVA using a within-subjects comparison of “acoustic decibel (dB)” showed a significant effect of treatment on startle response [F (1, 49) = 4.01, *p* < 0.01] (Figure 5C). After further analyzing the effect of treatment on startle response, an independent sample *t*-test revealed that seizure-exposed mice had decreased startle responses at the no stimulation compared to control mice [t (51) =6.1, *p* < 0.05] and 75 dB [t (51) =5.0, *p* < 0.05], although seizures significantly increased the startle response compared to controls at 120 dB [t (51) =1.9, *p* < 0.05]. There was also an interaction in genotype over startle response [F (1, 49) = 1.99, *p* < 0.05]. After further analyzing the effect of genotype on startle response, an independent sample *t*-test revealed that Fmr1 KO mice had increased startle responses at the no stimulation compared to control mice [t (51) =2.2, *p* < 0.05] and 75 dB [t (51) =2.7, *p* < 0.05], 85 dB [t (51) =2.3, *p* < 0.05], and decrease in startle response at 110 dB [t (51) =2.1, *p* < 0.05]. There was no between-subjects effect of genotype [F (1, 49) = 0.08, *p* = 0.78], treatment [F (1, 49) = 0.63, *p* = 0.43], or a two-way interaction of genotype and treatment on startle response over varying decibels [F (1, 49) = 0.21, *p* = 0.65]. 

### 3.6. Early-Life Seizures Increase Contextual Recall While Tone-Cued Recall Is Increased within Fmr1 KO Mice

**Figure 6:**
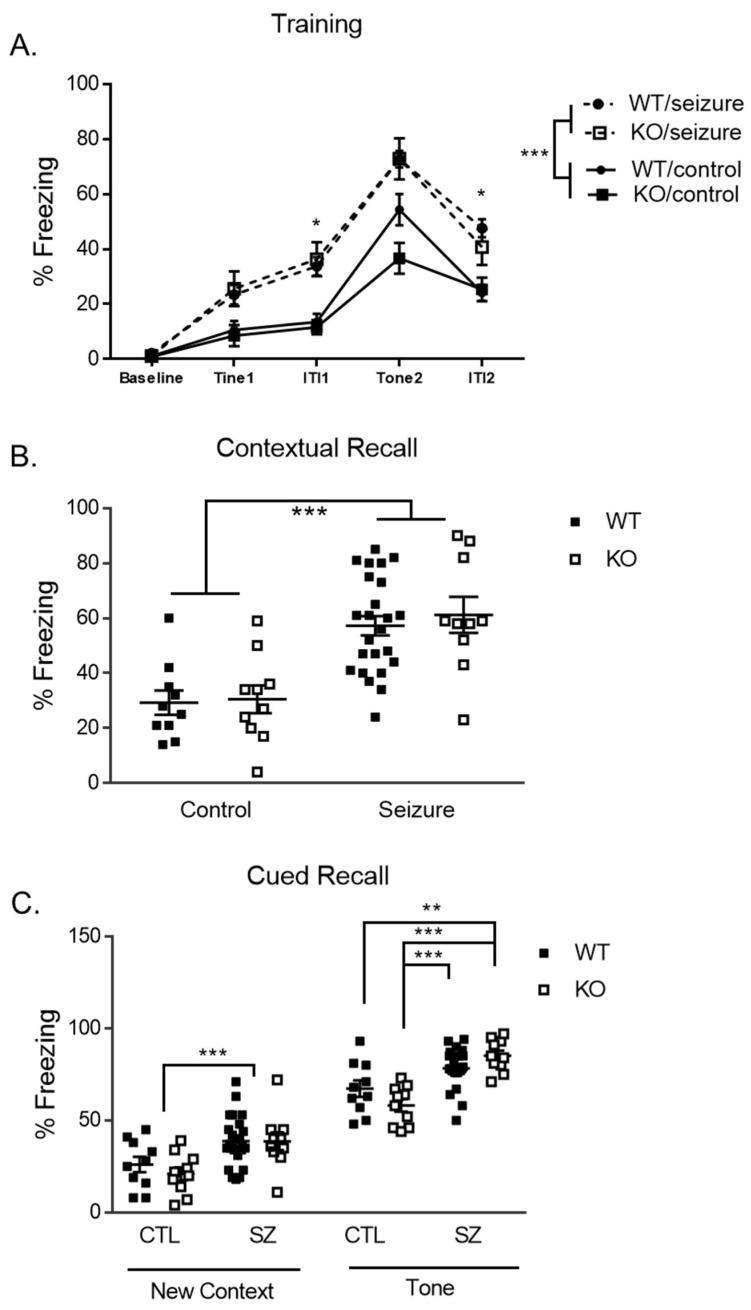
**Delay fear conditioning assay**. During training, seizure mice froze significantly more than control mice across the entire period, as well as specifically during the first intertrial interval (ITI1) and during the second ITI (**A**). During part 1 of testing on day 2 (contextual fear conditioning), seizure mice froze significantly more than control mice, with no effect of genotype (**B**). During part 2 of testing on day 2 (new context fear conditioning), seizure mice froze significantly more than control mice, with no effect of genotype (left panel (**C**)). There were several interactions during the conditioned stimulus portion of the delay fear conditioning. The Fmr1 knockout (KO) mice spent more time freezing compared to the control WT and KO mice. The WT mice with seizures spent more time freezing compared to the control KO mice. Data are expressed as mean ± standard error of the mean (SEM), * = *p* < 0.05, ** = *p* < 0.01, *** = *p* < 0.001. Each square represents a single subject. The WT mice are in solid squares and the KO mice are in open squares.

Day 1 of delay fear conditioning is a training day in which the mice are introduced to pairings of a conditioned stimulus (CS) and an unconditioned stimulus [22]. A two-way repeated measures ANOVA was utilized to analyze the data and the within-subjects factor of each trial and intertrial interval (baseline, tone 1, intertrial interval 1, tone 2, and intertrial interval 2). There was a within-subjects effect of treatment [F (4, 200) =9.71, *p* < 0.001], but no genotype [F (4, 200) = 1.35, *p* = 0.25] or a three-way interaction [F (4, 200) = 2.14, *p* = 0.078]. The between-subject effect of treatment was also significant [F (1, 50) = 29.48, *p* < 0.001], with an increase in freezing for the seizure mice (Figure 6A). Further analysis using independent *t*-tests of the effect of treatment on freezing revealed that seizures resulted in a higher rate of freezing across all tone 1 [t (52) = 3.2, *p* < 0.05], during the first intertrial interval [t (52) = 5.24, *p* < 0.05], tone 2 [t (52) = 5.2, *p* < 0.05], and the second intertrial interval [t (52) = 4.72, *p* < 0.01] compared to controls (Figure 6A). There was no main effect of genotype [F (1, 50) = 0.572, *p* = 0.45] or a treatment by genotype interaction [F (1, 50) = 0.36, *p* = 0.55]. 

Day 2 measured contextual fear conditioning, where freezing behavior was measured following being placed in the same context from the previous day for 5 min. Our two-way ANOVA showed a significant main effect of treatment [F (1, 50) = 11.41, *p* < 0.001], with seizure mice having expressed more freezing behavior for contextual memory (Figure 6B). 

The second phase of testing on day 2 examined cued recall. The freezing behavior of the mice was first measured in a novel context for 3 min, followed by 3 min where a cued associative fear memory stimulus (tone) was presented. A two-way ANOVA was used to examine the percent freezing at the mean baseline within the new context and a main effect of treatment was found [F (1, 50) = 14.82, *p* < 0.001], with seizures resulting in a higher percent freezing compared to controls (Figure 6C). For the cued recall portion of the test, we found a main effect of treatment [F (1, 50) = 34.38, *p* < 0.001], with mice who had undergone seizures showing a higher percentage of freezing when the tone was presented. There was no main effect of genotype [F (1, 50) = 0.10, *p* = 0.76]. There was also a two-way interaction between treatment and genotype on the percent freezing within the time points where the tone is present [F (1, 50) = 6.15, *p* < 0.05]. With this significant interaction, the animals were subdivided into four groups for a post hoc analysis: Fmr1 WT/Control, Fmr1 KO/Control, Fmr1 WT/seizure, and Fmr1 KO/seizure. Least Significant Difference (LSD) multiple comparisons showed that seizures in KO mice had a higher percent freezing by comparison to control KO mice (*p* < 0.001) during the presentation of the tone (CS) in the second phase of day 2. 

### 3.7. Early-Life Seizures Do Not Alter mTOR Signaling, Neural Inflammation, Scaffolding, or Ion Channel Protein Levels in Our Model Long-Term

Western blot analysis was done to examine the proteins associated with the downstream effects of the mTOR signaling pathway, neuroinflammation, and neuronal and synaptic excitability following early life seizures (ELS) in FXS and is presented in Table 1. Phosphorylated ribosomal protein S6 (S6) and protein kinase B (AKT) levels within the hippocampus were analyzed to observe changes in mTOR signaling. We found a significant increase in the ratio of pAKT/total AKT for genotype [F (1, 28) = 5.4, *p* < 0.05], whereby the KO mice had higher levels than the WT mice. We did not find any other differences in treatment or any interactions. We did not find any statistically significant differences in total AKT, phosphorylated AKT, total S6, phosphorylated S6, or pS6/total S6 levels. We did not find any other statistically significant differences in genotype, treatment, or genotype × treatment interaction for IBA1, GFAP, HCN1, HCN2, Kv4.2, or PSD-95 (Table 1).

## 4. Discussion

Two-hit models can be used to examine the impact of two separate insults on the developing brain. For our study, we examined the impact of early-life seizures on the genetic condition Fragile X Syndrome (FXS). Our study revealed that the superimposition of a high seizure load on Fmr1 KO mice resulted in an increased latency to the first nose poke and decreased anxiety-like behavior. Deletion of the FMR1 gene in these mice resulted in more repetitive and less anxiety-like behavior compared to WT mice. Seizures decreased social behavior in the social partition test, decreased locomotor activity across behavioral tasks, increased habituation to a auditory stimulus, and significantly increased contextual and cued freezing. Our Western blot analyses indicated a significant increase in phosphorylated AKT in the hippocampus of Fmr1 KO compared to WT mice, but no other differences in synaptic scaffolding proteins, ion channels, or inflammatory response markers were found. This study provides insight into the long-term behavioral and molecular impacts of seizures superimposed on Fmr1 KO mice.

Repetitive and stereotypic behavior is a core deficit of ASD. In a previous study where we induced status epilepticus (continuous seizures) in Fmr1 KO mice on PD10, we found an increase in consecutive nose pokes [21]. We did not find the same behavior because of flurothyl seizures, but repetitive behaviors may be more sensitive to superimposing seizures in Fmr1 KO mice. Future studies could use complementary tests in repetitive behaviors in order to clarify the impact of early-life seizures (ELS) through kainic acid, flurorthyl, or other chemoconvulsants and measure their influence on grooming, nestlet building, marble burying, and other tests that measure repetitive behaviors. 

Aberrant repetitive behavior is a robust behavioral phenotype in the KO mice. We found that KO mice expressed more repetitive behavior through increased consecutive nose pokes, reduced latency to perform their first nose poke, and an increase in the number of clockwise revolutions in the open field. Increased clockwise revolutions in KO mice were consistent with the findings of a prior study using Fmr1 KO on the FVB background [21]. An increase in revolutions has been found utilizing Fmr1 KO mice of the FVB strain [23] and in another study that used the C57BL/6J background strain [24]. Both C57 and FVB strains appear to support the impact of deletion of FMR1 on repetitive behavior. 

ELS resulted in alteration in repetitive behavior in the form of decreased grooming in the open field and increased latency to the first nose poke. However, the effect of ELS appears to produce different effects compared to the genotype effects and when ELS is induced in the Fmr1 KO. The effect of ELS is different from a previous paper with a similar seizure induction in WT C57BL/6J mice that found no effects on repetitive behavior [20]. Other rodent and seizure-induction models have shown a significant increase in repetitive behavior such as revolutions in the open field [25,26] and preservation through lever press preference [10]. Our decrease in repetitive behavior following ELS suggests that repeated seizures early in development may lead to a less repetitive phenotype. Further clarity on the repetitive tendency following ELS could be of interest in future studies. 

Anxiety is a common attribute of those with FXS [27]. In the current study, we found that the superimposition of a high seizure load on Fmr1 KO mice resulted in decreased anxiety-like behavior. Fmr1 KO mice have previously been found to express increased anxiety-like behavior [28]. The KO mice in the current study displayed lower anxiety-like behavior in the open field, spending more time in the center of the open field arena. We found no significant difference in the elevated plus maze, making the alteration in behavior not robust. Previously, decreased anxiety-like behavior within KO mice in the elevated plus maze has been observed [29,30,31,32]. Decreased anxiety-like behavior in the open field as described in multiple Fmr1 KO models is consistent with our findings [31,33,34]. Our findings along with previous literature suggest that Fmr1 KO mice may not robustly replicate the anxiety phenotype commonly found in humans with FXS. 

In contrast, we saw an overall increase in anxiety-like behavior following seizures in the open field task, but no difference in open-arm activity in the elevated plus maze. Our previous study using the same ELS model and strain found no differences between seizure and control mice in the elevated plus maze [20]. Previous investigations of anxiety-like behavior in rats showed that rats exposed to pilocarpine spent less time in the open arms of the elevated plus maze and less time in the center of the open field [25,26]. The difference in anxiety-like effects found may be attributed to differing induction models and alternate rodent models. The contrast builds our understanding of how different seizure types can impact anxiety-like behavior compared to our current ELS model. We found anxiety-like behavior in the open field task, but not in the elevated plus maze, making it difficult to suggest that KO mice are expressing a more anxiety-like phenotype. Future studies could potentially look further into the relationship between anxiety and locomotor activity.

Altered social behavior is a core deficit of ASD that has commonly been diagnosed in FXS individuals [27,35] and has been similarly reported in Fmr1 KO mice [36,37]. The current study found no differences in social behavior in KO and WT mice in the presence of an unfamiliar or familiar mouse. However, we did replicate our previous findings where ELS reduces social behavior. In a prior study, ELS-exposed mice were less social in the presence of a novel mouse and found less preference for the other mouse during the three-chamber task [20]. Induction of SE using KA or pilocarpine has been utilized in WT and a TSC haploinsufficiency rat model, another genetic model of ASD [25,26,38], and found that seizure induction results in decreased social exploration, contact social behavior, increased social evasion, and interaction with social novelty. The impact of ELS on social behavior is a robust finding. It is clear from this study and previous studies that ELS results in social behavior deficits, but it is not clear that the Fmr1 KO has a consistent social behavior deficit.

As the leading heritable cause of intellectual disability [27], FXS presents with learning and memory deficits that can be measured in KO mice [39,40,41]. In the present study, KO mice had increased freezing behavior in response to a tone. This behavior is suggested to indicate increased amygdala-based fear learning. KO mice also responded similarly to WT mice in the assessment of contextual fear conditioning. These results do not support prior work showing a deficit in amygdala-based fear memory manifesting as decreased freezing in Fmr1 KO mice across C57BL/6J and FVB background strains [39,40]. Others have found no significant effect in C57BL/6J mice [41,42]. The increased freezing behavior in our study may be driven by a decrease in overall locomotor activity or a generalized fear response. Contextual memory deficits, which are hippocampus-based, have previously been found in Fmr1 KO mice in the C57BL/6J strain [39] as well as in the FVB strain [43]. Our absence of hippocampal learning deficits does corroborate previous literature that did not identify memory deficits in the FVB KO [21,43] or C57BL/6J strain KO [41,42]. 

Individuals with FXS and those who have experienced ELS present with deficits in pre-pulse inhibition (PPI) and exaggerated acoustic startle reflexes due to hypersensitivity to sensory input [27,44,45]. The acoustic startle response and pre-pulse inhibition has been shown to be one of the most robust behavioral phenotypes of Fmr1 KO mice; however, the direction of the behavioral deficit has not always been consistent. There were differences between the Fmr1 KO and WT mice on the non-stimulus, 75 dB, 85 dB, and 110 dB in the PPI test. There have been mixed results regarding PPI in Fmr1 KO mice. Previous literature has found the opposite of what is observed in humans, with enhanced PPI [34], and others have mimicked humans with decreased PPI [46]. Some studies have also seen similar or minimal within-subject effects across genotypes at specific decibels within the task [33,47,48]. Previously in the FVB KO strain, we found increased PPI [21]. Further investigation would elucidate the connection between altered PPI and FXS. 

ELS have also been known to increase hypersensitivity to sensory input and dysregulate the startle response [49]. However, we saw improved habituation to the auditory stimulus following ELS across all trials and an increase in the startle response across most trials. Following status epilepticus (SE) in the same mouse strain, pre-pulse inhibition was unaffected [21]. In contrast to our findings, rats have shown deficits in sensorimotor gating following repeated seizure exposure [49,50]. We can propose that ELS results in a decibel-specific elevated startle response following ELS at a threshold of 100 dB. ELS appear to mitigate the reflexes to auditory stimuli on the first day of testing. Few studies have previously investigated habituation following ELS, suggesting that further utility of habituation in the presence of ELS could provide a more comprehensive perspective on the effects seizures have on sensorimotor gating in response to auditory stimuli.

The Fmr1 KO mouse has elevated translation in the hippocampus and the cortex and Fragile X Messenger Ribonucleoprotein 1 (FMRP) is localized to dendrites, dendritic spines, axons, and cell bodies [17]. Hyperactive signaling in the mTOR pathway through phosphorylation of S6 and Akt has been observed in FXS individuals post-mortem [51] and in animal models of epilepsy [52,53]. Hyperactive mTOR has been shown to be important to the seizure susceptibility reported in Fmr1 KO mice [17]. We previously found that mTOR signaling was increased in mice that received status epilepticus on postnatal day 10 [54]. We hypothesized that ELS would further increase mTOR signaling in the Fmr1 KO mice. Furthermore, we hypothesized that the elevated mTOR signaling would impact dendritic ion channels such as Kv4.2 and HCN channels. In our study, we found that Fmr1 KO mice had significantly elevated levels of phosphorylated AKT, but no difference in phosphorylated S6. The elevated phosphorylation of Akt and S6 found in prior studies examining mTOR activity in the hippocampus of FVB Fmr1 KO mice, using Western blotting [55,56] is believed to be driven by increased PI3K signaling [55]. Increased phosphorylation of S6 has been observed in the C57BL/6J strain of the Fmr1 KO mouse in the frontal cortex and neocortex [17,57]. This may suggest regional dependent mTOR hyperactivation within this mouse model. We did not find alterations in dendritic ion channels, microglia activation, or astrocyte activation, which have been found in other studies where mice have hyperactive mTOR signaling [14,15,16]. It has been previously shown that hyperactive mTOR signaling can result in an increase in Kv4.2, HCN channels, microglia activation, and astrocyte levels [14,16]. It may be that a higher level of mTOR activation, such as when there is genetic deletion of phosphatase and tensin homologue, which is an important negative regulator of the mTOR pathway, is needed in order to result in such alterations in the brain. Future studies could further examine alterations in ion channels, microglia, and astrocytes in Fmr1 KO mice. 

## 5. Conclusions

Limited studies have investigated genetic mutations and how they are impacted by seizure induction, including a previous study looking at SE in the Fmr1 FVB KO mice aimed to utilize such a model [21]. Our study revealed that the superimposition of a high seizure load on *Fmr1* KO mice resulted in an increased latency to the first nose poke and deceased anxiety-like behavior. Changes in repetitive behavior demonstrate how seizures induced during early life may impact one of the core components of autism. However, superimposing seizures on a mouse model of Fragile X Syndrome resulted in minor behavior deficits and no additional molecular changes. 

## Figures and Tables

**Table 1:** Densitized values for mTOR, neuroinflammatory proteins, and dendritic ion channels.

Target	Group			
Protein Levels	WT/CTL	WT/SZ	KO/CTL	KO/SZ
pS6/Total S6	100 ± 1.6	95.04 ± 2.0	98.56 ± 6.1	104.56 ± 2.5
pAKT/Total Akt	100 ± 6.7	96.99 ± 6.6	117.50 ± 20.5	129.71 ± 30.2
GFAP	100 ± 2.69	115.97 ± 11.6	115.54 ± 13.2	119.10 ± 18.6
Iba-1	100 ± 9.2	115.35 ± 18.7	123.20 ± 39.3	96.49 ± 11.4
HCN1	100 ± 12.4	93.32 ± 13.7	82.55 ± 15.4	109.35 ± 30.1
HCN2	100 ± 16.0	95.39 ± 13.6	151.06 ± 42.2	72.74 ± 13.2
Kv4.2	100 ± 10.6	109.31 ± 6.7	104.31 ± 18.9	105.43 ± 16.7
PSD-95	100 ± 4.0	124.23 ± 16.5	102.32 ± 11.9	117.25 ± 8.2

Values represent the mean ± standard error of the mean.

## Data Availability

The original data presented in the study are openly available at https://doi.org/10.6084/m9.figshare.26485282.v1, accessed on 27 August 2024..

## Supplementary Materials

The following supporting information can be downloaded at: https://www.mdpi.com/article/10.3390/brainsci14090892/s1, Supplemental S1: full statistical results.
